# A Diagnostic Dilemma: Catastrophic or Seronegative Antiphospholipid Syndrome

**DOI:** 10.7759/cureus.18745

**Published:** 2021-10-13

**Authors:** Christina D Enescu, Brinda Basida, Nirav Zalavadiya, Rayhaan Akram, Housam Sarakbi

**Affiliations:** 1 Rheumatology, Wayne State University School of Medicine, Detroit, USA; 2 Internal Medicine, Detroit Medical Center Sinai-Grace Hospital, Detroit, USA; 3 Rheumatology, Wayne State University Detroit Medical Center, Detroit, USA

**Keywords:** rheumatology, autoimmune disease, systemic lupus erythematosus, seronegative catastrophic antiphospholipid syndrome, seronegative antiphospholipid syndrome, catastrophic antiphospholipid syndrome, antiphospholipid syndrome

## Abstract

Catastrophic antiphospholipid syndrome (CAPS) and seronegative APS (SN-APS) are rare and severe variants of antiphospholipid syndrome (APS). Due to the significant morbidity and mortality associated with these variants, early recognition and adequate treatment with immunomodulatory agents and anticoagulation are crucial. Here, we report a rare presentation of seronegative CAPS in a young adult with systemic lupus erythematosus (SLE) who presented with seizures, encephalopathy, and quadriplegia. Brain imaging revealed intracranial hemorrhage and attenuated vessels in the Circle of Willis suggestive of vasculitis. Imaging also revealed bilateral pulmonary emboli involving the main pulmonary, segmental, and subsegmental arteries; lower extremity deep vein thrombosis in the right common femoral vein; and superficial venous thrombi in the left cephalic and basilic veins. Due to the absence of APS seropositivity and the catastrophic nature of her presentation, namely the widespread thrombi formation and multiorgan involvement, there was high suspicion for a diagnosis of seronegative CAPS. After two weeks of high doses of immunomodulatory agents, plasmapheresis, and intravenous immune globulin (IVIG) treatment, the patient showed clinical improvement and a reduced burden of venous thrombi. The predicament of not being able to use anticoagulation in this patient due to cerebral hemorrhage added to the complexity and uniqueness of this case.

## Introduction

Antiphospholipid syndrome (APS) is a rare autoimmune disorder characterized by vascular thrombosis and persistently positive antiphospholipid antibodies (aPL): lupus anticoagulant (LAC), anticardiolipin antibody (aCL), and/or anti-β2-glycoprotein-I antibody (aβ2GPI) [1].

Catastrophic APS (CAPS) is the most severe form of APS in which widespread thrombi, usually microthrombi, rapidly form in various organs [1]. CAPS is unique in that it can cause multiple organ dysfunction syndromes, systemic inflammatory response syndrome, and unusual organ involvement, and carries a high mortality rate despite therapy.

Seronegative APS (SN-APS) is another variant of APS in which multiple episodes of thrombosis occur in the absence of cardiovascular risk factors or an identifiable cause of thrombosis [2]. Its presentation is suggestive of a thrombophilic condition, like APS, but in the absence of aPL positivity on at least two occasions. According to the most recent studies, antibodies against phosphatidylethanolamine, phosphatidic acid, phosphatidylserine, phosphatidylinositol, vimentin/cardiolipin complex, and annexin A5 can be useful in testing for SN-APS [2]. However, there is a lack of standardized assays to measure the levels of these antibodies for use in routine clinical practice.

Here, we describe a young patient with severe systemic lupus erythematosus (SLE), presenting with multiorgan thrombi formation, exhibiting features of APS despite aPL seronegativity, highly suggestive of seronegative CAPS. In this report, we aim to highlight the rarity of the disease and the uniqueness of this case so it can be identified early and treated.

## Case presentation

An 18-year-old African-American female with a history of hidradenitis suppurativa presented to the hospital for new-onset seizures. During admission, the patient demonstrated worsening encephalopathy, quadriplegia, aphasia, seizures, and right eye blindness. Labs showed high-titer positive antinuclear antibody (ANA) (1:1280), anti-Smith antibody (Anti-Sm Ab) (114 AAU/mL), anti-double-stranded DNA antibody (anti-ds DNA Ab) (1:320), and low levels of C3 and C4 complement, thus confirming the diagnosis of SLE. However, labs were negative for LAC, aCL, and aβ2GPI.

**Figure 1:**
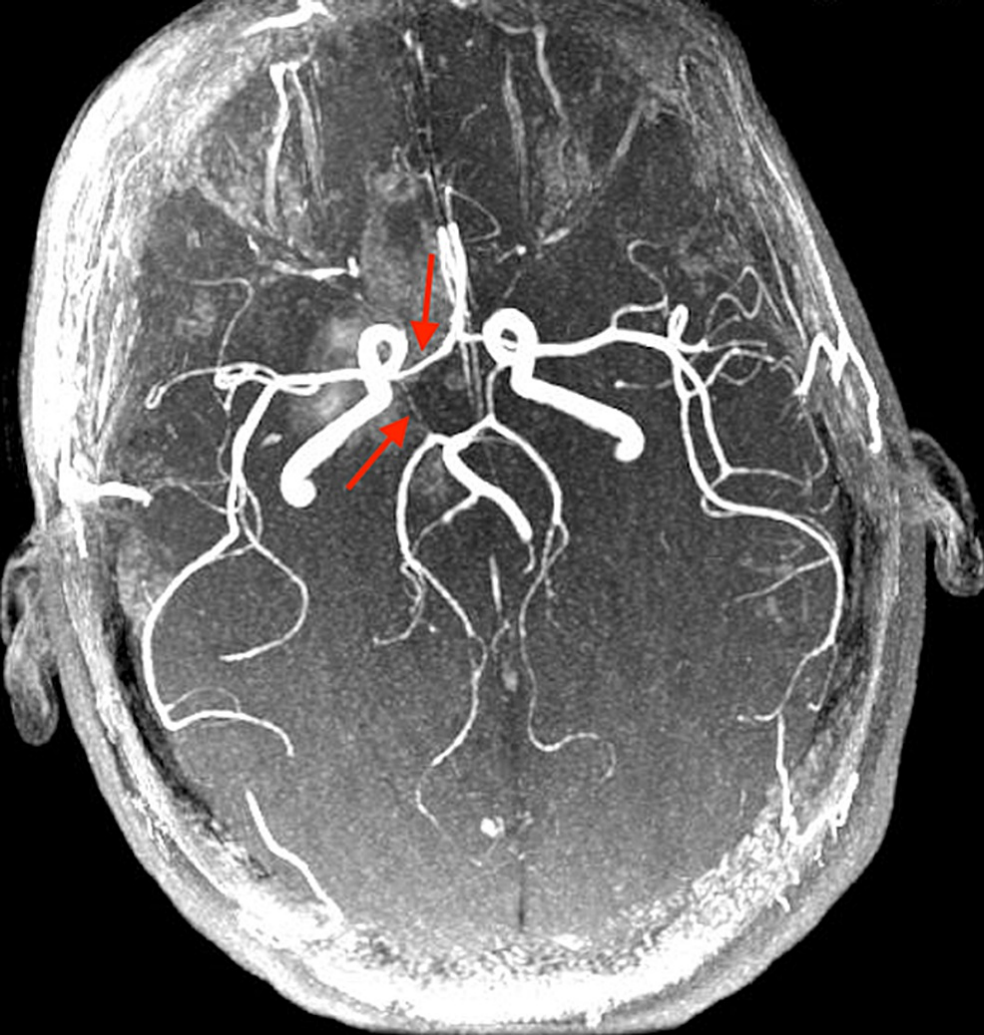
Magnetic resonance angiography (MRA) of the brain shows attenuated vessels with a beaded appearance in the Circle of Willis (red arrows).

Imaging revealed several central nervous system (CNS) hemorrhages present in the frontal lobes, temporal lobes, basal ganglia, and brainstem. Magnetic resonance imaging (MRI) with magnetic resonance angiography (MRA) and magnetic resonance venography (MRV) showed intraparenchymal hemorrhage, partial thrombosis of the left transverse sinus, and attenuated vessels with a beaded appearance in the Circle of Willis (Figure 1); these findings were concerning for vasculitis. Findings on the fundoscopic exam suggested central retinal artery occlusion, central retinal vein occlusion, and bilateral retinal vasculitis.

The patient was treated with pulse steroids, cyclophosphamide, IVIG, and rituximab for suspected SLE-induced CNS vasculitis. Upon clinical improvement after three weeks, she was then transferred to a rehab institute, where she became tachycardic and hypoxic during one session of physical therapy. Computed tomography (CT) of the chest, CT-PE, at this time showed bilateral pulmonary emboli involving the main pulmonary, segmental, and subsegmental arteries. Venous duplex ultrasound revealed a deep vein thrombosis in the right common femoral vein and superficial venous thrombi in the left cephalic and basilic veins.

After transfer to the ICU, the patient experienced a new onset of tachycardia and tachypnea. Physical exam showed anisocoria in which the right pupil was larger than the left, sluggish reaction to light of the right pupil, the chronic hyperpigmented discoid appearance of bilateral auricles, hair thinning, and weakened strength in the bilateral upper and lower extremities (1/5). Lab findings showed leukopenia, anemia, and thrombocytopenia. Coagulation studies including Protein C and S and anti-thrombin deficiency and COVID-19 test were unremarkable. Anti Jo-1, anti-Scl 70, anti-SSA, anti-SSB, and anti-RNP antibodies were negative.

Due to concern for CAPS, the patient was given intravenous (IV) pulse steroids (methylprednisolone 1g daily for three days), followed by 1 mg/kg oral steroids, and initiated on plasmapheresis. Anticoagulation was held due to the recent intracranial hemorrhage. Despite this, the patient had a further decline in her C3 and C4 complement levels. Given the severe systemic burden of clots, negative lab values for aPL, and now with the continued decline in C3 and C4 complement despite multiple immunomodulatory agents, seronegative CAPS was highly suspected as the diagnosis.

Therefore, possible eculizumab administration was discussed and plans were made to obtain it. During this process, the patient was continued on her immunosuppression regimen consisting of cyclophosphamide infusion (1g every four weeks), IV methylprednisolone (1mg/kg daily), and plasmapheresis (five sessions total). This regimen resulted in gradual clinical improvement and an increase in C3 and C4 levels. CT of the thorax revealed a reduced pulmonary embolism burden. She was continued on high-dose steroids, cyclophosphamide for a total of six doses, and plasmapheresis for five sessions. Due to her improving clinical stability, eculizumab and systemic anticoagulation were held off, and the patient was sent to rehabilitation and discharged.

## Discussion

APS is characterized by vascular thrombosis and/or pregnancy complications with persistently positive aPL antibodies: LAC, aCL, and/or aβ2GPI [1]. SN-APS has been proposed to involve antibodies that are not currently tested in routine clinical practice and therefore patients are seronegative for the above aPL antibodies. Catastrophic APS (CAPS) involves at least three or more organs, systems, or tissues; development of symptoms simultaneously or in less than one week; histopathologic evidence of small-vessel occlusion; and the presence of aPL. Definite CAPS diagnosis is confirmed when all four criteria are met [1].

It is a challenge to distinguish seronegative CAPS, from other conditions that also involve multi-organ thrombosis. Such conditions include disseminated intravascular coagulation (DIC), heparin-induced thrombocytopenia, and thrombotic microangiopathies [3]. Unlike CAPS, DIC is usually associated with bleeding and an underlying systemic disorder, as well as prolonged prothrombin time (PT) and activated partial thromboplastin time (aPTT), which were absent in our patient. Thrombotic microangiopathies typically have thrombocytopenia and specific lab abnormalities related to the underlying cause, both of which were not seen in our patient.

We suspect our patient had seronegative CAPS for several reasons. Her history of SLE and subsequent widespread thrombi formation with multi-organ dysfunction, in the absence of other systemic illnesses, is consistent with APS; her lack of aPL seropositivity in the setting of an APS-like presentation is consistent with SN-APS. Moreover, the severity of her presentation, specifically the acute burden of thrombosis within a short time frame despite immense immunosuppression, is highly suggestive of CAPS. Though CAPS is usually associated with thromboses in multiple small vessels rather than large vessel deep vein thromboses or stroke [4], the latter can occur as it did in our patient. In addition, she eventually responded well to further immunosuppression with additional courses of plasmapheresis and cyclophosphamide, both of which are known treatments for CAPS [5,6]. Given these points, seronegative CAPS is highly suspicious for this young patient.

We found six cases of seronegative CAPS reported in the literature [5-10]. One patient was a 61-year-old woman with rapidly recurrent arterial and venous thromboses and emboli leading to cerebral infarctions [8]. Her clinical findings and course were suspicious for CAPS and she was reported to have negative aPL, although she did have a history of lung cancer that may have had contributed to her prothrombotic state. Another case describes a 43-year-old woman who presented with a severe headache and was suspected of having an arachnoid hemorrhage. She was later found to have thrombi in the cerebral venous sinus, aorta, and spleen. She was treated in a manner similar to our patient, with steroids, plasmapheresis, and IVIG, although anticoagulation was used as well. One 17-year-old patient, similar in age to our patient, presented initially with SN-APS and was only found to have CAPS after anticoagulation withdrawal [10].

In some rare cases of CAPS or APS, aPL may become transiently negative at the time of thrombosis due to the consumption of these antibodies during the thrombo-occlusive episode [1]. However, the aPL antibodies would become positive after the acute episode. The hypocomplementemia, namely low C3 and C4, that our patient exhibited can be observed in both APS and SLE [11]. Complement levels are frequently used to trend disease severity in SLE. Given her degree of immunosuppression with her burden of thrombosis, and the lack of other SLE manifestations, the hypocomplementemia in our patient was felt to be due to an APS-like cause.

CAPS is a rare but life-threatening condition. Terminal complement activation plays an important role in amplifying the aPL-induced organ damage via thrombophilia and endothelial cell activation, thus leading to progressive systemic thrombosis. Terminal complement blockade has been suggested as a selective approach in further preventing the progression of the microthrombosis cascade [12]. Eculizumab, a humanized monoclonal antibody, targets against the terminal complement C5. It blocks the cleavage of C5 and prevents the production of proinflammatory and prothrombotic molecules C5a and membrane attack complex C5b-9 [13]. This drug has shown positive outcomes in few cases of massive aPL-induced complement activation [12], so it was under consideration for use in our patient.

## Conclusions

In general, physicians should have a low threshold for diagnosis of SN-APS or CAPS in lupus patients due to its major complications and increased mortality. Due to the overlapping features of both SLE and APS, both conditions should be screened for upon suspicion of either diagnosis. This case report documents the findings of an 18-year-old patient with lupus who presented with features of both CAPS and SN-APS, suggesting the rare diagnosis of seronegative CAPS. The strikingly severe presentation of this patient and added limitation of not being able to use an anticoagulant made the management of this case unique and challenging. With this case, we hope to contribute to the existing body of knowledge of seronegative CAPS so that this condition can be properly recognized and treated.
